# A case study of the Philippines’ COVID-19 after action review

**DOI:** 10.5365/wpsar.2024.15.3.1113

**Published:** 2024-07-23

**Authors:** Elliot Brennan, Charade B Mercade-Grande, Rowena Capistrano, Yui Sekitani, Kathleen Ryan, Landry Ndriko Mayigane

**Affiliations:** a Health Security Preparedness Department, World Health Organization, Geneva, Switzerland.; b Department of Health, Manila, Philippines.; c World Health Organization Representative Office for the Philippines, Manila, Philippines.

Health system performance depends on continual learning. (*1*) The need for such learning is most visible during and after protracted health emergencies. After action reviews (AARs) are one tool for health system learning. A 2019 systematic review of AARs noted that the tool “contributes to a culture of continuous personal, collective and institutional learning aimed at the gathering of lessons learned and best practices.” (*2*) Key features of AARs include effective leadership engagement, the equal participation of team members, the inclusion of stakeholders, a positive and safe environment for feedback, and the generation of collective knowledge. (*2*-*4*) When effectively employed, AARs can lead to evidence-based improvements in health emergency preparedness, response and resilience.

On 5 May 2023, the Director-General of the World Health Organization (WHO) lifted the status of public health emergency of international concern from COVID-19 upon the recommendation of the International Health Regulations (2005) Emergency Committee. The report by the Emergency Committee recommended that “States Parties should update respiratory pathogen pandemic preparedness plans [by] incorporating learnings from national and subnational after action reviews.” (*5*)

Following this recommendation, WHO developed the *Guidance for conducting a country COVID-19 after action review (AAR)*. (*6*) The Guidance was reviewed during consultations with both internal and external experts. In June 2023, the Philippines became the first country to conduct a COVID-19 AAR using WHO’s new methodology. A draft of the Guidance and tools were used to design the Philippines’ COVID-19 AAR. Following the AAR, a writing workshop was conducted in July 2023 to translate the AAR’s findings into the Philippine Pandemic Preparedness, Response and Resilience Plan.

## PROCESS

The Philippines’ COVID-19 AAR was coordinated by the Secretariat of the Inter-Agency Task Force for the Management of Emerging Infectious Diseases, a body that was reactivated by the executive government on 28 January 2020 to respond to the COVID-19 outbreak. The Inter-Agency Task Force is an intersectoral collaborative body established in 2014 to prevent, prepare for and respond to outbreaks of emerging infectious diseases in the Philippines.

A 2-day in-person COVID-19 AAR (15–16 June 2023), followed by a 2-day in-person writing workshop (6–7 July 2023), were both held in Manila, Philippines. Focus groups and key informant interviews were conducted to provide further depth and breadth to insights about cross-cutting aspects. Multiple agencies were represented, including from the private sector, academia and the medical sector, as well as offices of the government. Throughout the process, working groups included representatives from diverse agencies.

The overall objectives of the process were to review and document the lessons learned about the Philippines' preparedness for and response to the COVID-19 pandemic, to identify corrective actions and policies implemented, and to inform the drafting of the Philippine Pandemic Preparedness, Response and Resilience Plan.

### COVID-19 after action review

The Philippines’ COVID-19 AAR was based on the Philippines National Action Plan against COVID-19 following WHO’s methodology. (*6*) The scope and methods proposed in the Guidance were adapted to meet the country’s needs and to align with its response, based on its prevent, detect, isolate, treat, reintegrate and vaccinate (PDITR+V) strategy: (*7*)

prevent – implement health promotion and ensure adherence to public health and social measures;
detect – implement surveillance, testing and contact tracing;
isolate and quarantine – monitor quarantine facilities at all levels;
treat – rely on the patient referral system, hospital readiness and case management;
reintegrate – ensure economic recovery, social healing, awareness of possible reinfection, minimum health standards; and
vaccinate.

During working group discussions, emphasis was placed on selected pillars from the WHO Guidance:

country-level coordination, planning and monitoring;
national legislation and financing;
mass gatherings;
infection prevention and control;
operational support and logistics for managing supply chains and ensuring workforce resilience; and
risk communication and community engagement, and infodemic management.

Each multisectoral working group identified the challenges, impacts and enabling or limiting factors encountered during the COVID-19 pandemic. The working groups then recommended strategic actions for both the short-term (12–24 months) and long-term (5 years). These were ordered by priority and prepared for use in the second phase of the process, the writing workshop.

### Writing workshop

The writing workshop was designed according to the draft Asia Pacific Health Security Action Framework, (*8*) replacing the Asia Pacific Strategy for Emerging Diseases and Public Health Emergencies, (*9*) which enabled it to incorporate lessons learned from the COVID-19 pandemic. The draft Framework was later endorsed by Member States in the Western Pacific Region. (*10*)

The multisectoral writing groups prioritized addressing the strategic actions from the AAR discussions to develop up to 10 activities to be undertaken during the next 12–24 months. Working groups explored the feasibility (difficult or easy) and impact (high or low) of each activity, which allowed them to be prioritized. Implementation pathways were mapped for each activity. Barriers to completing each activity were discussed and mitigation measures were identified, as well as policy pathways that could help overcome barriers. To facilitate implementation, performance indicators and completion deadlines were also identified for each proposed activity.

## Discussion

During the AAR process, participants cited the importance of the multiagency nature of discussions, which allowed for systems thinking and overall ownership for planning and implementing strategic actions going forward. Participants also appreciated the flexibility of the discussions, which allowed for deep exploration of root causes and corrective actions.

The prioritization of activities and identification of barriers to implementation were two core elements of the writing workshop that helped reveal clear policy pathways for the short-term action plans. Participants reflected on how prioritizing enabled them to reduce an otherwise overwhelming number of potential activities to a more manageable number of time-bound and impactful ones. During the working groups, some participants noted feeling empowered by identifying barriers to implementation. They noted the value of charting a course to remove known and foreseeable bottlenecks.

Reflecting on the process, one participant noted that everybody wants to talk about their experiences and best practices, but organizing input and writing are quite a challenge. The COVID-19 AAR and writing workshop provided a pathway to address these obstacles. The process allowed for the capture and codification of knowledge and for collaborative translation into proposed policies. Insights from the COVID-19 AAR and writing workshop are being transformed into an actionable Philippine Pandemic Preparedness, Response and Resilience Plan. The lessons identified may in turn inform other planning and preparedness workstreams, such as supporting the Joint External Evaluation process, the States Parties Self-Assessment Annual Report and National Action Plans for Health Security.

The Philippines’ COVID-19 AAR is a model for conducting future AARs on COVID-19 and other protracted health emergencies. The authors encourage countries to employ WHO’s methodology for COVID-19 AARs and to share their experiences to ensure intercountry learning.
